# Role of Mediterranean diet in the development and recurrence of meningiomas: a narrative review

**DOI:** 10.1007/s10143-023-02128-8

**Published:** 2023-09-22

**Authors:** Roberta Costanzo, Irene Simonetta, Sofia Musso, Umberto Emanuele Benigno, Luigi Maria Cusimano, Evier Andrea Giovannini, Kevin Giardina, Vincenzo Abrignani, Irene Baglio, Alessio Albanese, Domenico Gerardo Iacopino, Rosario Maugeri, Antonino Tuttolomondo

**Affiliations:** 1 Neurosurgical Clinic, AOUP “Paolo Giaccone”, Post Graduate Residency Program in Neurologic Surgery, Department of Biomedicine, Neurosciences and Advanced Diagnostics, School of Medicine, University of Palermo, 90127 Palermo, Italy; 2 Internal Medicine and Stroke Care Ward, Department of Promoting Health, Maternal-Infant Excellence and Internal and Specialized Medicine (ProMISE) G. D’Alessandro, University of Palermo, Piazza delle Cliniche n.2, 90127 Palermo, Italy; 3 Department of Neurosurgery, Fondazione Policlinico Universitario “A. Gemelli” IRCCS, Università Cattolica del Sacro Cuore, 00168 Rome, Italy

**Keywords:** Meningioma, Mediterranean diet, Healthy lifestyle, Brain tumor, Prevention

## Abstract

Several studies through the years have proven how an unhealthy nutrition, physical inactivity, sedentary lifestyle, obesity, and smoking represent relevant risk factors in cancer genesis. This study aims to provide an overview about the relationship between meningiomas and food assumption in the Mediterranean diet and whether it can be useful in meningioma prevention or it, somehow, can prevent their recurrence. The authors performed a wide literature search in PubMed and Scopus databases investigating the presence of a correlation between Mediterranean diet and meningiomas. The following MeSH and free text terms were used: “Meningiomas” AND “Diet” and “Brain tumors” AND “diet.” Databases’ search yielded a total of 749 articles. After duplicate removal, an abstract screening according to the eligibility criteria has been performed and 40 articles were selected. Thirty-one articles were excluded because they do not meet the inclusion criteria. Finally, a total of 9 articles were included in this review. It is widely established the key and protective role that a healthy lifestyle and a balanced diet can have against tumorigenesis. Nevertheless, studies focusing exclusively on the Mediterranean diet are still lacking. Thus, multicentric and/or prospective, randomized studies are mandatory to better assess and determine the impact of food assumptions in meningioma involvement.

## Introduction

Healthy nutrition and lifestyle have certainly a crucial role in the prevention of cardiovascular, chronic-degenerative, immune-inflammatory, and tumor-related diseases: according to the World Cancer Research Fund, adopting a healthier lifestyle could have an impact on the onset of 3–4 million cancer cases worldwide.

The term Mediterranean diet, proposed by Keys et al., was based on the observations of a lower rate of cardiovascular disease in the countries bordering the Mediterranean Sea (Cyprus, Greece, and Italy) than in the Netherlands, the USA, and Finland [1] Typical features of Mediterranean diet are a high consumption of vegetables, legumes, fresh fruit, non-refined cereals, nuts, and olive oil, in association with a moderate intake of fish, milk products and ethanol, and red wine consumed during the main meals; lastly, according to the diet, red meat consumption is limited to lowered quantities [2].

Over the years, several studies have shown how a balanced diet could represent an effective preventive measure, proving that an unhealthy nutrition, physical inactivity, sedentary lifestyle, obesity (consequence of a healthy or unhealthy lifestyle), and smoking constitute relevant risk factors in cancer onset [3–5].

Even though the association between cancer and diet has been extensively debated, emphasizing how nutrition plays an essential role, both negatively or positively, in cancer development and/or progression, there are not enough studies focusing on its role and effect on cerebral tumors, in particular on meningiomas.

### Aims

This study aims to present an overview about the relationship between meningiomas and the Mediterranean diet and whether the Mediterranean diet can be useful in meningioma prevention or in the prevention of their recurrence.

## Sources

### Study selection

We performed a broad literature search in PubMed investigating the presence of a correlation between Mediterranean diet and meningiomas. Recorded studies were exported to Mendeley software. Only articles in English language were included. All the duplicates were removed, and a manual search was also performed to identify eventual additional studies of the reference sections. Two reviewers (U.E.B. and L.M.C.) independently screened the titles, abstracts, and full manuscripts; then, the results were analyzed and combined. The following MeSH and free text terms were identified “Meningiomas” AND “Diet” (24 articles) and “Brain tumors” AND “diet” (725 articles).

Taking into account the first MeSH term, an evaluation of studies with a temporal range from 1983 to 2022 was performed. For what it concerns the second MeSH term, the temporal range was from 1962 to 2022.

### Eligibility criteria

The articles were selected according to the following *inclusion criteria*:

- Full articles in English
- Studies regarding Mediterranean diet, although not directly mentioned but containing foods from the Mediterranean diet
- Studies regarding meningiomas

The following are the* exclusion criteria*:

- Studies about diet others than Mediterranean one
- Studies reporting other brain tumors, without mentioning or talking about meningiomas
- Articles not in English language
- Studies including animals
- Studies regarding the pediatric population

According to the criteria above, all articles were identified independently by two reviewers (U.E.B. and L.M.C.) and then the results combined. The extracted data included authors, year, study design, number of patients included, type of food evaluated, the aim of the article, and the results obtained by the authors.

Our search yielded a total of 749 articles. After duplicate removal, an abstract screening according to the aforementioned eligibility criteria has been performed, and 40 articles were selected. Thirty-one articles were excluded because they do not meet the inclusion criteria. A total of 9 articles were included in the present review.

In the studies included, 509513 patients were evaluated. Among them, 7540 (1,47%) were affected by malignant brain tumor, 943 (12,5%) by meningiomas and, finally, 2637 (34,97%) were healthy controls. Study characteristics are summarized in Tables 1 and 2.

**Table 1:** Summary of the studies including nutrients belonging to the Mediterranean diet associated to brain tumor risk

Authors	Year	Country	Study design	No. of patients	Primitive tumor	Food	M/F ratio	Age	Aim	Results
Boeing et al. [6]	1993	Germany	Case–control study	196 patients affected by brain tumor + 518 controls	115 (gliomas) + 81 (meningiomas)	Potatoes, vegetables, vegetable juice, fruit, fruit juice, cheese, brown bread, processed meat, fish	99 men and 127 women	25 to 75	Investigation of the effect of the ingestion of N-nitroso precursor on the development of gliomas and meningiomas	-Intake of processed meat it is related to significant positive association with gliomas and meningioma -Intake of nitrosamines showed high risk to develop meningiomas
Guo et al. [7]	1994	China	No data	1983	No data	Salt-preserved vegetables, green vegetables, carrot, fruit, eggs	No data	No data	Associations between primary brain tumor mortality and dietary habits	Etiologic clues about the relationship between primary brain tumors and dietary intake
Kaplan et al. [8]	1997	Israel	Case–control study	139 patients affected by brain tumor	59 (malignant tumors) and 80 (benign tumor, primarily meningiomas)	No data	No data	18 to 75	Evaluation of influence of nutritional factors on tumor proliferation	-Positive association between high protein intake and brain tumor -High intake of cholesterol and sodium inversely related to brain tumor
Hu et al. [9]	1999	China	Case–control study	129 patients affected by brain tumor + 258 controls	73 (gliomas), 56 (meningiomas)	Cereals, potatoes, Chinese cabbage, onion, garlic, fresh vegetables, fruit, meat, soya, fresh fish, salted fish, salted vegetables	56 meningiomas (65 males, 64 women)	20 to 74	Evaluation of the possible role of diet in the etiology of adult brain tumors	High consumption of fresh vegetables, fruit, and foods rich in vitamin E may decrease brain cancer risk
Bansal et al. [10]	2002	India	Observational study	20 affected by brain tumor	20 brain tumors	Cured meat, vegetables, fruits, vitamin A, fish	No data	No data	Determine the effects of lifestyle and dietary habits in the development of brain tumor	Stressful and sedentary lifestyle, wrong diet, alcohol addiction, and smoking habit relate to higher brain cancer risk
Dimitropoulou et al. [11]	2008	UK	Case–control study	637 patients affected by brain tumor + 876 controls	436 gliomas + 201 meningiomas	Dietary food intake, vitamins, and other food supplement	No data	18 to 69	To test the hypothesis that increased dietary Zn intake is protective against brain tumor development	-Dietary Zn intake is correlated to a significant risk reduction for meningioma -No correlation between dietary Zn intake and gliomas
Terry et al. [12]	2009	USA	Meta-analysis	No data	Gliomas (1185), meningiomas (332)	Cruciferous vegetables, leafy green vegetables, yellow-orange vegetables, cured meat, noncured meat, fresh fish, eggs, grains, citrus fruit	0:4	42 to 58	Evaluate associations between histology-specific risk and consumption of specific food groups	Selected dietary food groups may be associated with adult gliomas and its subtypes but not with meningiomas
Allès et al. [13]	2016	France	Case–control study	494 patients affected by brain tumor, 985 controls)	201 neuroepithelial tumors, 193 meningiomas, 100 other CNS tumors	Vegetables, fruits, grilled meat, and poultry, processed meat and smoked fish, fresh fish, aspartame-containing food, all alcoholic beverages	206 M 288 W	No data	Relationship between the consumption of food and the occurrence of tumors in the CNS	Higher fruit and vegetable intake was inversely associated with meningiomas
Lian et al. [14]	2017	USA	Meta-analysis	501,617	4428	Fish	No data	No data	Clarify the association between fish intake and brain tumor risk	Fish intake might be associated with lower risk of brain cancer

**Table 2:** Association between food and risk of brain tumors

Food	Meningiomas (risk)	Gliomas (risk)
Satured fat	+	-
Fish	P.F	P.F
Leafy green vegetables	-	P.F
Yellow-orange vegetables	-	P.F
Cured meat	-	-
Noncured meat	-	+
Eggs	-	+
Cheese	P.F	-
Citrus fruit	-	+
Grains	-	+
Salt-preserved vegetables	+	+
Onion	P.F	P.F
Fruit	P.F	P.F
Poultry	P.F	P.F
Salted fish	+	+
Potatoes	-	P.F
Brown bread	-	P.F

The positive sign indicates an increased risk of developing a brain tumor (meningioma or glioma); the negative sign indicates the absence of correlation between food and brain tumor risk; P.F. (protective factor) highlights the inverse relationship between food and brain tumor (meningioma or glioma)

It must be noticed that not univocal results can be obtained due to data fragmentation and lack of specific data about tumor histotypes.

## Discussion

### The Mediterranean diet: its role against cancer

Tumors represent the second leading cause of death worldwide; they recognize complex multifactorial pathophysiological mechanisms, including oxidative stress, chronic inflammation, alterations in cell cycle regulation, and dysregulation of pro-oncogenes, which result from the combination of endogenous and exogenous conditions. Among the environmental factors, one of the most important is represented by nutrition, which can influence, both negatively or positively, some cellular and molecular characteristics related to cancer.

The Mediterranean diet is probably the best known healthy dietary pattern, characterized by a high intake of antioxidant and anti-inflammatory nutrients, including olive oil (as the main source of fat) and vegetables (fruits, legumes, nuts, seeds, and whole grains); a moderate intake of fish, white meats, eggs, dairy products, and red wine; and reduced consumption of red meats. Its protective effects are well documented in several chronic diseases, such as atherosclerotic cardiovascular diseases, diabetes mellitus, neurodegenerative and respiratory diseases, and depression, as well as for tumors, especially colon, breast, and prostate [15–17].

Over the years, research focused on mechanisms through which the Mediterranean diet plays a protective role in tumor development, protecting cells from the mechanism involved in tumorigenesis, such as oxidative and inflammatory processes, DNA damage, neoplastic angiogenesis, and spreading of metastases [15, 18].

The main components of the Mediterranean diet are fruits and vegetables, olive oil, fish, and red wine; they contain bioactive nutrients that counteract cell degeneration and proliferation of cancerous cells, thanks to their antioxidant and anti-inflammatory properties, such as polyphenols, flavonoids, carotenoids, fiber, and monounsaturated fatty acids [19–21].

*Polyphenols*, the main bioactive components of the Mediterranean diet, have known anti-inflammatory, antioxidant, chemoprotective, and pro-apoptotic effects and act with complex and multiple mechanisms, including epigenetic variations, transcriptome, and expression proteins that modulate different signaling pathways (MAPK, PI3K-Akt, and Wnt/β-catenin activated by oxidative stress), thus reducing proliferation, cell migration and invasion, and angiogenesis [22–24]. Polyphenols can also modulate microRNAs, which regulate the expression of tumor-suppressing oncogenes or genes leading to a reduction in tumor growth and its metastatic potential [25, 26].

*Flavonoids* promote the elimination of pollutants (heavy metals, polycyclic aromatic hydrocarbons, dioxins, pesticides, ultra-particulate) from tissues or mitigate their effects [27].

Several nutrients of the Mediterranean diet can modify the epigenome (which regulates the modulation of gene expression as studied by nutrigenomics), inhibiting the development of tumors and acting as protective factors [28]; this effect can be partly explained by the ability to support an effective repair of DNA damage, modulating the activity of histones and RNA methyl marker [29]. A high adherence to the Mediterranean diet has a protective role against metabolic and oxidative DNA damage, improving the antioxidant system, as demonstrated by the reduced levels of oxo-708-dihydro-20-deoxyguanosine and the increase in those of HDL-cholesterol and glutathione peroxidase [30].

Many natural compounds contained in the Mediterranean diet counteract angiogenesis, which plays an essential role in tumor growth and in the development of metastases. The Mediterranean diet inhibits the development of the metastatic phenotype in cancer cells and, also, alterations in physiological bone remodeling [31, 32].

### Literature review of the most prestigious studies about cancers and Mediterranean diet

At the end of the 1950s, the “Seven Countries of Cardiovascular Diseases Study” enrolled, at entry, 16 population cohorts in eight nations of seven countries for a total of 12,763 men (aged 40–59); the aim of this epidemiological study was to highlight dietary cultural contrasts and to compare cardiovascular disease rates, related to diet differences. The findings revealed by this study allowed to point the so-called “Mediterranean diet” and showed a significant protective effect of this dietary pattern on tumors [33].

Since then, several retrospective case–control studies refined this concept and documented on a larger scale the beneficial effects of this dietary pattern in tumor prevention and progression through some parameters and scores, such as the Food Frequency Questionnaire (FFQ) and the alternate MD score (aMED). Among them, strong evidence come from the European Prospective Investigation into Cancer and Nutrition Study (EPIC study), which included 476,160 subjects recruited in 10 European countries from 1991 to 2001, with a mean follow-up of 13.9 years. In a systematic review of 110 high-quality studies on the EPIC cohort, fruit and vegetable consumption showed a protective effect against colorectal, mammary, and pulmonary neoplasms, and fruit only showed a protective effect on prostate cancer; high fish consumption was related to a lower risk of colorectal cancer (and fatty fish with lower risk of breast cancer), and calcium and yogurt intake was protective against colorectal and prostate cancer. Overall, adherence to the Mediterranean diet was protective against colorectal and mammary neoplasms [34].

Another example is given by the Women’s Health Initiative Observational Study, which included 86,090 women with a mean follow-up of 17 years and proved that high levels of aMED score were protective for the development of squamous cell carcinomas [35]. In the Nurses’ Health Study, which enrolled 29,474 women, patients most adherent to aMED showed a lower risk of colorectal adenoma, especially of high-risk subtypes [36]. In an average 18-year follow-up of 2966 participants in the Framingham Offspring Study, women with moderate or high adherence to the Mediterranean diet showed a 25% lower risk of neoplasms than those with lower adherence, and the benefits were even better in normal weight subjects; the association was weaker in men, except in non-smokers [37].

### Prospective studies and Mediterranean diet

The protective role of Mediterranean diet in tumor prevention is then well established; however, less is known about the role of diet in tumor progression in patients with an already established diagnosis of neoplasm [38]. In this regard, in a prospective study of 80 patients affected by colorectal cancer, Acevedo et al. found that high adherence to the Mediterranean diet was associated with less severe histological degrees and lower presence of synchronous adenomas [30]. Moreover, in the Multiethnic Cohort Study were enrolled 6370 patients, finding a better aMED scores associated with lower risks of mortality and cancer [39].

### Mediterranean diet and meningiomas

Spotlight on brain tumors, meningiomas represent the most common primary intracranial tumors, and they overall account about 36% of all CNS lesions [40]. They are usually benign lesions (WHO I), showing a slow and non-infiltrating growth, whereas only about 19% are histologically aggressive and rapidly growing (WHO II & III). Incidence is age-related (peak at 75–84 years old age group) and about threefold higher in females (2.27:1 F:M ratio) [41, 42].

Thus far, the only well-recognized epidemiologic risk factors for these tumors are the exposure to ionizing radiation and genetic syndromes (such as the neurofibromatosis II); over the years, several foregoing epidemiologic researches have provided partial evidence in a correlation between dietary habits—in particular nitrites and nitrates exposure—and an increased risk of developing primary brain tumors, including meningiomas [43].

N-Nitroso compounds (NOCs) are distributed among food in different concentrations in the form of as N-nitrosamines and N-nitrosamides, secondary amines and amides which, endogenously, can be transformed into some metabolites called ethylnitrosoureas, in the presence of nitrite [12, 44]. The largest dietary sources of nitrosamines have been noted to be cured meat, namely the addition of salt, sugar nitrite and/or nitrate, and other compounds to meat-based products for the purpose of preservation, appearance, and flavor improvement. Overall, these molecules are deemed to be potential carcinogens in the development of brain tumors, based on evidence from animal studies [12, 44, 45].

Quite the opposite, the nutritional intake of nitrosation inhibitors, like vitamins C and E, which are commonly found in some fruits and vegetables, may relate to a reduced risk of developing the above-mentioned tumors [42].

This awareness is suggested by different studies. Kaplan et al., indeed, in a case control study (59 malignant primary brain tumors and 80 meningiomas) have showed that high protein intake is strictly related to brain tumor development [8]. Preston-Martin et al. [46], in several population-based case–control series, found an increased risk in brain meningioma development in women with high level consumption of nitrite-cured meat, even though the same correlation appeared unclear in a population composed only by men [47]. The same authors also found that the assumption of citrus fruit was slightly protective against meningiomas [48]. Similarly, Burch et al. [49]suggested a protective role of fruits, but not of vegetables, in the genesis of primary brain tumors; they did not find a significant relationship with the nutritional intake of processed meat. The role of vitamins C and E in brain’s tumorigenesis inhibition was also confirmed by Mirvish [50] and Hu et al. [9], even if Boeing et al. [6] did not confirm the aforementioned association in their series. Moreover, Huncharek and Kupelnick [51] in their meta-analysis, alongside with previous epidemiologic studies, suggested a possible correlation between maternal consumption of cured meat and an increased risk of pediatric brain tumor development.

Finally, Dimitropoulou et al. [11], in a case control study, highlighted that a diet rich in zinc seems to be related to a lower risk of meningioma development, even if a correlation with glioma has not been proved yet.

In order to stick on the subject of food intake and correlative risk of developing brain tumors, previous literature on this field has proven that also metabolic disorders are strongly linked to the development of primary brain tumors.

Currently, as far as our knowledge goes, there is substantial evidence in the role of obesity and poor physical activity in increasing meningioma risk [10, 52–54]: obesity is associated with the development of type II diabetes and so higher circulating levels of IGF-1 and insulin, which can pass through the blood–brain barrier and thus promote, through several mechanism, meningioma’s growth and progression [52–57] Moreover, a higher concentration of adipose tissue is deemed to be associated with a major blood concentration of estrogens, which could therefore lead to the development and progression of meningiomas. Interestingly, this awareness could partially explain the higher incidence of these tumors among the female gender [53].

The results of our research are consistent with the hypothesis that carcinogens contained in some categories of food, and the amount of food itself may be involved into brain tumor etiology, suggesting that a combination of N-nitroso compounds (NOCs) and other supplements consumed from diet may be involved with brain tumors’ carcinogenesis.

## Conclusions

It is widely accepted the key and protective role that a healthy lifestyle and a balanced diet can have against tumorigenesis. The Mediterranean diet contains many nutrients counteracting angiogenesis and safeguarding from tumor development. Currently, several studies have discussed its protective role focusing on colorectal, mammary, and pulmonary tumors, not extensively mentioning primary brain tumor. According to preliminary results retrieved from this overview, some categories of food, belonging to the Mediterranean diet, have shown a protective role in brain’s tumorigenesis. Nevertheless, papers focused just on the Mediterranean diet are still lacking.

Thus, multicentric and/or prospective and randomized studies are mandatory to better assess and determine the impact of dietary intake in meningioma risk.

## Data Availability

Not applicable.
